# Alkalinity to calcium flux ratios for corals and coral reef communities: variances between isolated and community conditions

**DOI:** 10.7717/peerj.249

**Published:** 2014-02-06

**Authors:** Liana J.A. Murillo, Paul L. Jokiel, Marlin J. Atkinson

**Affiliations:** 1 University of Hawaii, Hawaii Institute of Marine Biology, Kaneohe, Hawaii, USA

**Keywords:** Coral reef, Biogeochemistry, Alkalinity anomaly, Reef ecology, Alkalinity flux, Ocean acidification, pH balance, Corals, Macroalgae, Sedimentary diagenesis

## Abstract

Calcification in reef corals and coral reefs is widely measured using the alkalinity depletion method which is based on the fact that two protons are produced for every mole of CaCO_3_ precipitated. This assumption was tested by measuring the total alkalinity (TA) flux and Ca^2+^ flux of isolated components (corals, alga, sediment and plankton) in reference to that of a mixed-community. Experiments were conducted in a flume under natural conditions of sunlight, nutrients, plankton and organic matter. A realistic hydrodynamic regime was provided. Groups of corals were run separately and in conjunction with the other reef components in a mixed-community. The TA flux to Ca^2+^ flux ratio (*Δ*TA: *Δ*Ca^2+^) was consistently higher in the coral-only run (2.06 ± 0.19) than in the mixed-community run (1.60 ± 0.14, *p*-value = 0.011). The pH was higher and more stable in the mixed-community run (7.94 ± 0.03 vs. 7.52 ± 0.07, *p*-value = 3 × 10^−5^). Aragonite saturation state (*Ω*_arag_) was also higher in the mixed-community run (2.51 ± 0.2 vs. 1.12 ± 0.14, *p*-value = 2 × 10^−6^). The sediment-only run revealed that sediment is the source of TA that can account for the lower *Δ*TA: *Δ*Ca^2+^ ratio in the mixed-community run. The macroalgae-only run showed that algae were responsible for the increased pH in the mixed-community run. Corals growing in a mixed-community will experience an environment that is more favorable to calcification (higher daytime pH due to algae photosynthesis, additional TA and inorganic carbon from sediments, higher *Ω*_arag_). A paradox is that the alkalinity depletion method will yield a lower net calcification for a mixed-community versus a coral-only community due to TA recycling, even though the corals may be calcifying at a higher rate due to a more optimal environment.

## Introduction

Accurate calcification measurements of coral reef organisms and communities are needed if we are to understand the carbon cycle on reefs and to predict and monitor the effects of ocean acidification (OA) on these crucial coastal ecosystems. Most laboratory experiments evaluate response of coral colonies isolated from other ecosystem components such as sediment and algae. This study was directed at comparing the metabolic response of isolated corals to the response of corals in the presence of other ecosystem components.

During the last few decades the alkalinity depletion method (Kinsey, 1978; Smith & Kinsey, 1978) has been the primary means of measuring net calcification rate on corals and coral reefs. Total alkalinity (TA) can be defined as the total buffering capacity of the seawater, or the excess of proton acceptors over proton donors (Dickson, 1981):

(1) \begin {eqnarray}\label {eq1} \mbox {TA}&=&[\mbox {HCO}_{\mbox {3}}^{{-}}]+2[\mbox {CO}_{\mbox {3}}^{\mbox {2-}}]+[\mbox {B(OH)}_{\mbox {4}}^{{-}}]+[\mbox {OH}^{{-}}]+[\mbox {HPO}_{\mbox {4}}^{\mbox {2-}}]\nonumber \\ &&+\,2[\mbox {PO}_{\mbox {4}}^{\mbox {3-}}] +[\mbox {SiO(OH)}_{\mbox {3}}^{{-}}] +[\mbox {NH}_{\mbox {3}}]+[\mbox {HS}^{{-}}]-[\mbox {HSO}_{\mbox {4}}^{{-}}]\nonumber \\ &&-\,[\mbox {H}^{\mbox {+}}]_{\mbox {F}} -[\mbox {HF}] -[\mbox {H}_{\mbox {3}} \mbox {PO}_{\mbox {4}}]+[\mbox {minor acids} - \mbox {minor bases}] \end {eqnarray}

TA is influenced predominantly by bicarbonate and carbonate ion concentration along with a myriad of other minor compounds (Eq. (1)). A simple relationship exists between TA and calcification when coral metabolism is measured in isolation from other coral reef components (coral in a beaker with no sediment, nutrient or algae). Under these conditions the release of protons by calcification lowers TA (Smith & Key, 1975). The basic reaction can be described as follows:

(2) \begin {equation}\label {eq2} \mbox {Ca}^{2+}+\mbox {CO}_{2} +\mbox {H}_{\mbox {2}} \mbox {O}\Leftrightarrow \mbox {CaCO}_{3} +2\mbox {H}^{+} \end {equation}

The above equation shows that two protons are produced for every mole of CaCO_3_ precipitated with the consequent reduction of TA by two moles for every mole of CaCO_3_ produced. The reverse reaction occurs during dissolution. This is the basis of the alkalinity anomaly technique that has been widely used to measure calcification rates. Photosynthesis and respiration alter pH but do not change TA (Smith & Key, 1975).

Chisholm & Gattuso (1991) validated the alkalinity depletion technique for corals by comparing change in total alkalinity (*Δ*TA) to change in Ca^2+^ concentration (*Δ*Ca^2+^) by incubating a colony of the coral *Pocillopora damicornis* in a beaker containing filtered seawater. They determined the *Δ*TA:*Δ*Ca^2+^ ratio to be 2.0 as predicted by Eq. (2). However, TA can be altered through other coral reef processes such as organic matter production from photosynthesis (Kim & Lee, 2009), anaerobic diagenesis in sediments (Emerson & Hedges, 2003; Thomas *et al.*, 2009; Mackenzie *et al.*, 2011), and nutrient transformation (Brewer & Goldman, 1976; Wolf-Gladrow *et al.*, 2007) as shown by Eq. (1). Microbes living within the plankton and sediment can also make a contribution to TA in shallow water systems (Brewer & Goldman, 1976; Boucher *et al.*, 1998; Kim, Lee & Choi, 2006; Werner *et al.*, 2008). The net effect these non-coral biogeochemical processes will generally increase TA over time, causing the *Δ*TA:*Δ*Ca^2+^ ratio to decrease below 2.0.

Changes in TA caused by such interactions between components of mixed reef communities (coral, plankton, sediments, and algae) need further investigation. The metabolic response of isolated components versus response in mixed communities can differ. The possible limitations of the alkalinity depletion technique due to mixed-community processes other than coral calcification were recognized at the outset (Kinsey, 1978; Smith & Kinsey, 1978). Some reefs may depart from the theoretical *Δ*TA:*Δ*Ca^2+^ ratio of 2.0 because of processes such as release of ammonia and sulfide during organic matter diagenesis as well as denitrification (Davies & Kinsey, 1973). Researchers have shown that the *Δ*TA:*Δ*Ca^2+^ ratio is not 2:1 in some systems (Andersson, Bates & Mackenzie, 2007). Thus we undertook a series of experiments to evaluate the change in the *Δ*TA:*Δ*Ca^2+^ ratio for the various components of the coral reef ecosystem (coral, plankton, sediments, and algae) incubated as individual components in comparison to coral mixed communities containing all of the components.

## Methods and Materials

**Figure 1:**
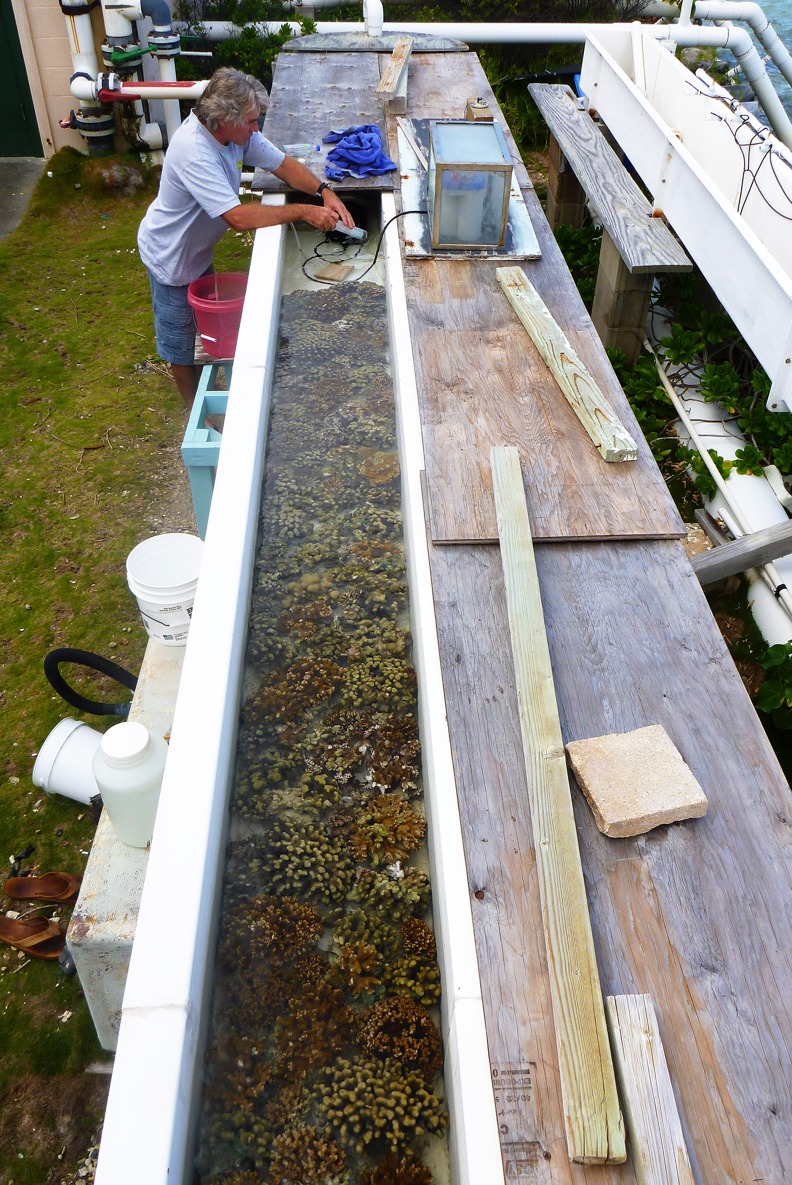
Flume system. The flume system with the shade cloths taken off showing the sub-sample bucket in use.

Experiments were conducted at the Hawaii Institute of Marine Biology (HIMB) in an outdoor flume (Fig. 1) located next to the Coconut Island Reef. The flume had a volume of 2.28 m^3^ and an air to seawater surface area of 8 m^2^. Water in the flume was re-circulated in a closed loop with a current of 10 cm s^−1^, which is comparable to the hydrodynamic regime of Kaneohe Bay reefs (Langdon & Atkinson, 2005). Irradiance in the shallow outdoor flumes was reduced by 50% with shade cloth in order to mimic reef conditions at greater depths (1–2 m). Sunlight, temperature and hydrodynamic flow were within the typical range found on Kaneohe Bay reefs. The flume was filled with unfiltered water from the HIMB sea water system that is pumped from a depth of 2 m off the Coconut Island windward reef. This water provided the organisms with realistic levels of plankton and nutrients from their natural environment. The water was aerated during all experiments in order to stabilize dissolved oxygen levels and keep the communities from becoming anoxic at night. During each “incubation” the inlet flow was turned off for 5 days while the flume motor continued recirculating the sea water over the corals at 10 cm s^−1^. The five day incubation time permitted the accurate measurement of Ca^2+^ flux. Salinity was measured throughout the experiment. Integrated samples were taken every other day over the 5 day static incubation using of a 20 L subsampling bucket (Fig. 1). Water from the recirculating flume was slowly siphoned through a 1 cm diameter tube into the bucket for a period of 10 min. Bottles were rinsed three times with sample water before being filled from the bucket, and the remaining integrated sample returned to the flume. Unfiltered samples were placed in glass bottles, sealed and stored at 25°C. Analysis was performed within 24 h. After the 5 day incubation period the flume was continuously flushed for 2 days with unfiltered seawater from the HIMB seawater system to refresh the supply of nutrients and plankton. This process was then repeated three times for each community in the flume. A 24 h study during which samples were taken every 2 h was conducted to obtain diel data needed to evaluate the pattern of calcification which is high during daylight due to light-enhanced calcification and low or negative at night due to dissolution.

Five flume incubations were performed: (1) the coral-only run with a substrate of 2 m^2^ of mixed corals, (2) the mixed-community run consisted of the same corals from the coral-only run, but with 2 m^2^ of fine carbonate sediment and 2 m^2^ of filamentous algae that had been allowed to develop on the flume walls, (3) the sediment-only run with the same 2 m^2^ sediment as in the mixed community, (4) the macroalgae-only run with 2 m^2^ of the marco-alga *Gracillaria salicornis* and (5) the plankton-only run with the unfiltered water in a clean flume without any other organisms. The plankton-only run can also be viewed as the blank control on the benthos. Temperature, pH, Ca^2+^ concentration and TA were measured concurrently. All incubations intrinsically include the microbial plankton community in the unfiltered seawater.

### Alkalinity flux to Ca^2+^ flux ratios for the coral-only versus mixed-community

TA and calcium were measured on the first, third and fifth days of each 5 d run. Concentrations were normalized to the starting salinity in order to correct for changes in concentration due to evaporation and precipitation. The *Δ*TA:*Δ*Ca^2+^ ratio was calculated in the coral-only run versus the mixed-community run to determine if the ratio deviated from the expected 2.0. The experimental design also included the sediment-only run, macroalgae-only run and the plankton-only run to allow interpretation of differences from the predicted *Δ*TA:*Δ*Ca^2+^ ratio in the coral-only run and the mixed-community run. Mean daily net calcification (G_net_) in mmoles CaCO_3_ m^−2^ d^−1^ was calculate for each 5 day run using initial and final TA values.

The coral species that were used in the coral-only run were selected as representative of a typical coral community found on the Coconut Island Reef in Kaneohe Bay, Oahu. The map area of the coral colonies in the flume (49% *Porites compressa*, 41% *Montipora capitata*, 9% *Pocillopora damicornis* and 1% *Fungia scutaria*) was estimated from vertical photographs of the flume benthos using pixel counts performed in Photoshop C4. On an aerial basis the flume contained 2 m^2^ (map area) of coral cover.

The sediment-only run contained sediment sufficient to cover 2 m^2^ of the flume bottom to a depth of 12 cm that was collected from a near shore back-reef community. This sediment consisted of fine, carbonate sands with a relatively high amount of organic matter and anoxic layering. The sediment was allowed to adjust to the flume for a week under conditions of flowing water at a rate of 10 cm s^−1^ before experimentation to allow natural anoxic diagenesis layers to reform after being disturbed in the collection process. The macroalgae-only run contained the alga *Acanthophora spicifera* that covered 2 m^2^ of the bottom. The plankton-only run consisted of a clean flume with no organisms other than what was contained in the seawater. This run can also be viewed as the seawater control. For the mixed-community run, the same corals used in the coral-only run were supplemented with carbonate sediment as described for the sediment-only run along with filamentous algae on the flume walls.

### Diel cycle

Seawater samples were taken over a 24 h period from the coral-only run at approximately 2 h intervals starting from 15:30 on 30 May 2012. Samples were taken from the mixed run at approximately 2 h intervals from 14:00 h on 27 June 2012. Salinity, pH, TA and Ca^2+^ concentration were determined from the samples with replicate analysis of each sample. Both diel studies were conducted during the summer months, one month apart from each other at the same time in the lunar cycle (1–2 days after first quarter moon).

### Measurements

TA was measured in duplicate by automatic titration with 0.1 N HCl solution on a Metrohm titrator, using Dickson standards for alkalinity (Dickson, Sabine & Christian, 2007). The samples were kept in a water bath of 25°C beforehand and placed in a water-jacketed container held at that temperature during analysis. Standard error (SE) for the measurement was 5 *μ*M alkalinity. Ca^2+^ and TA values were normalized to the starting salinity at the beginning of each incubation experiment. Salinity and pH were measured using an Orion conductivity probe calibrated to Dickson standards. Ca^2+^ was measured in duplicate with the standard titration of Ca^2+^ with EGTA and Ca^2+^ selective electrodes (Edmond, 1970). Measurement SE was ± 0.02 mM Ca^2+^. All fluxes in the experiments were at least four to five times the SE, and in most cases the magnitude of change was more than twenty times the SE. HCO$_{{3}}^{{-}}$, CO$_{{3}}^{{2-}}$, pCO_2_ and *Ω*_arag_ were calculated from salinity, temperature, TA and pH data using the CO2SYS program developed by Lewis and Wallace (1998). Average Kaneohe Bay phosphate and silicate concentrations along with constants from Mehrbach *et al.* (1973) refit by Dickson & Millero (1987) were used in the calculations as described in (Shamberger *et al.*, 2011).

**Table 1:** Alkalinity and Calcium Uptake Ratios. Uptake of TA and Ca^2+^ (mean ± SE) in coral-only run versus the mixed-community run during 5 day incubation with the resulting *Δ*TA:*Δ*Ca^2+^ ratio.

Community	Ca^**2+**^ uptake	TA uptake	**Δ**TA:**Δ**Ca^**2+**^ ratio
Coral-only run	228 ± 64	470 ± 126	2.06 ± 0.19
Mixed-community run	171 ± 30	274 ± 57	1.60 ± 0.14

Statistical testing was carried out using Origin Pro 8 Data Analysis and Graphing Software. Data are reported as mean ± SE of the mean. Significant differences between parameters were tested by using two-tailed student t-tests where H_o_ = no difference between mesocosms, significance at *P* < 0.05. If the *P* value was less than 0.05 for a 95% confidence level, the null hypothesis was rejected.

## Results

Average temperature was consistent for all experiments and ranged between 23.5°C and 24.0°C. Net calcification rates (Table 2) were within the normal range (170–230 mmol m^−2^ d^−1^) reported in the literature (Gattuso *et al.*, 1998; Langdon & Atkinson, 2005; Atkinson & Cuet, 2008; Andersson *et al.*, 2009). The calcification rate stayed consistently high for all trials containing corals, indicating that the corals remained in good health. After initial acclimation the corals did not show the stress response of excess mucous production and appeared to be healthy with extended polyps for feeding. There was no evidence of disease or bleaching.

### Alkalinity flux to Ca^2+^ flux ratios for the coral-only versus mixed community

**Figure 2:**
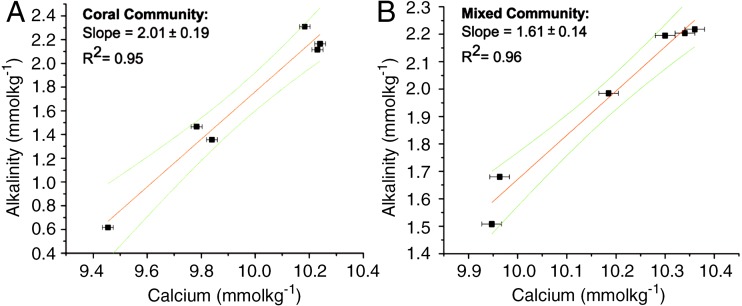
**ΔTA**: **ΔCa**^**2+**^ for coral and mixed communities. Change in TA and Ca^2+^ during the coral-only run (A) and mixed-community run (B). The slope of the line corresponds to the *Δ*TA: *Δ*Ca^2+^ ratio for each run. For the coral-only run, the slope is 2:01, *r*^2^ = 0.95 and for the mixed-community run the slope is 1.61, *r*^2^ = 0.96.

**Figure 3:**
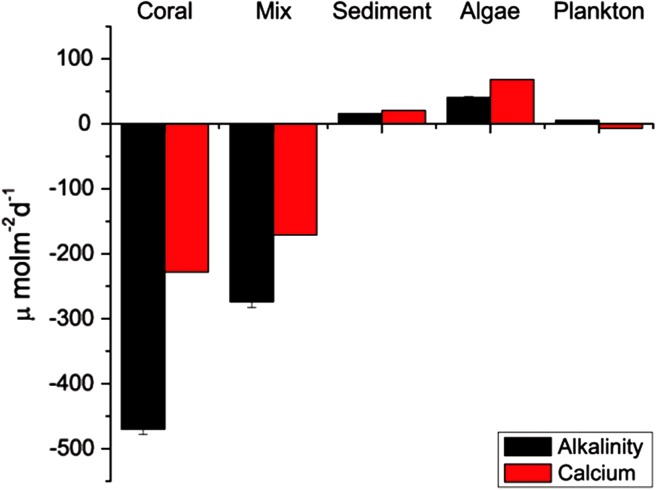
Average rates of TA and **Ca**^**2+**^ change. Change in TA and Ca^2+^ for each run in *μ*mol m^−2^d^−1^. Sediment, plankton and algae increased TA while corals decreased TA.

The ratios for the coral-only run and the mixed-community run were significantly different from each other (*p* < 0.05) (Table 1). The ratio of change between successive samples was also determined (*Δ*TA:*Δ*Ca^2+^ ratio) with a mean of 2.02 ± 0.05 for the coral-only run and 1.55 ± 0.09 for the mixed-community run, *p*-value for the difference in the ratios = 0.01. Additionally, the paired concentrations of TA and calcium for each sample was plotted and the corresponding slope (*Δ*TA:*Δ*Ca^2+^ ratio) showed very similar values: 2.01 ± 0.19 for corals and 1.61 ± 0.14 for the mixed-community (Fig. 2). Since the rate of Ca^2+^ uptake was the same for both runs, this offset (18%) indicates additional sources of TA to the water column (i.e., buffering) in the mixed-community run. These ratios, and the effect of the sediment and algae, can also be seen by looking at the overall fluxes of Ca^2+^ and TA for all incubations in Fig. 3.

**Table 2:** Summary table of chemical conditions. Mean ± SE for daytime measurements in the communities and community components. *P*-values indicate significance of difference between the coral-only run and mixed-community run.

	pH	Temp (°C)	Ca^**2+**^ (mmol kg^**−1**^)	TA (**μ**mol kg^**−1**^)	DIC (**μ**mol kg^**−1**^)	HCO_**3**_ (**μ**mol kg^**−1**^)	CO$_{{\boldsymbol {3}}}^{{\boldsymbol {2-}}}$ (**μ**mol kg^**−1**^)	CO_**2**_ (**μ**mol kg^**−1**^)	**Ω**_**arag**_	*n*	G_**net**_ mmol **m**^**−2**^ d^**−1**^
Coral	7.5 ± 0.07	23.8 ± 0.3	9.75 ± 0.0	1765 ± 6	1766 ± 60	1651 ± 59	72 ± 9	42 ± 6.2	1.12 ± 0.14	20	310
Mixed	7.9 ± 0.04	23.8 ± 0.3	10.05 ± 0.0	2015 ± 76	1802 ± 60	1622 ± 60	159 ± 12	20.5 ± 7.3	2.51 ± 0.20	14	210
*p*-value	3 × 10^−5^**	0.97	0.005*	0.023*	0.073	0.74	6 × 10^−6^ **	0.03*	2 × 10^−6^ **	-	-
Sediment	8.00 ± 0.02	24 ± 0.56	11.5 ± 0.21	2185 ± 17	1793 ± 31	1609 ± 31	172 ± 9.8	11.4 ± 0.78	2.73 ± 0.14	7	6.4
Algae	8.56 ± 0.13	23.5 ± 0.39	10.67 ± 0.0	2126 ± 56	1402 ± 139	1028 ± 181	370 ± 41	3.39 ± 1.8	5.89 ± 0.68	6	22.9
Plankton	8.05 ± 0.03	24.5 ± 0.54	10.4 ± 0.03	2153 ± 69	1933 ± 88	1707 ± 75	215 ± 28	10.45 ± 0.46	3.4 ± 0.43	4	2.8

* significant at 0.05 level.

** significant at 0.001 level.

### Water chemistry conditions 

**Figure 4:**
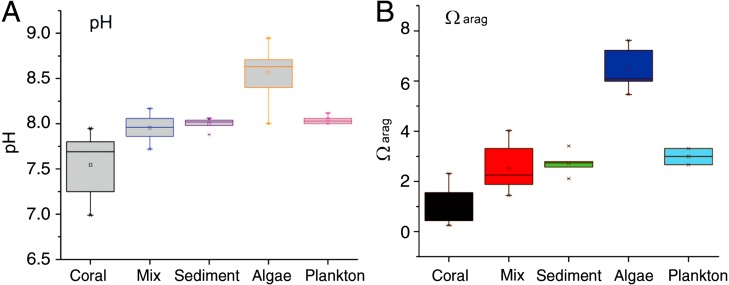
pH and *Ω*_**arag**_ for all communities. Range of daytime pH values for all five incubations (A) compared to the aragonite saturation states *Ω*_arag_ (B). Boxes contain 75% of the data, and the lines indicate the entire range.

Seawater pH in the mixed-community run was higher and more stable than the coral-only run (Fig. 4). Mean pH was 7.52 ± 0.07 for the coral-only run versus 7.94 ± 0.03 in the mixed-community run, *p*-value: 3 × 10^−5^. The coral-only run showed a mean *Ω*_arag_ of 1.12 ± 0.14 versus 2.51 ± 0.2 in the mixed-community run, *p*-value = 2 × 10^−6^ (Fig. 4). *Ω*_arag_ never fell below 1 for the mixed-community run. However, *Ω*_arag_ dropped below 1 for 30% of the samples in the coral-only run. Both incubations had the same corals, so these changes are not due to alterations in coral biomass or community composition. [Ca^2+^] was 9.75 ± 0.07 mmol kg^−1^ for the coral-only run and 10.05 ± 0.07 mmol kg^−1^ in the mixed-community run, *p*-value = 0.0054. Average CO_2_ concentration was twice as high in the coral-only run (42 ± 6.15 *μ*mol kg^−1^ compared to 20.5 ± 7.3 *μ*mol kg^−1^ in the mixed-community run, *p*-value = 0.03).

The sediment-only run showed the highest TA while the macro-algae run showed the highest pH (Table 2), indicating that these components are driving an increase in calcification above that of the coral only community by providing a more suitable habitat. This increase in coral calcification would have to be measured by buoyant weight or another method since the efflux of TA and Ca^2+^ out of the sediment detracts from the drawdown signal of these ions used to infer coral calcification.

### Component comparison

Results of the sediment-only run, macro-algae run and plankton run are shown in Table 2 in comparison to the coral-only run and the mixed-community run. The macro-algae run had the highest mean pH and the highest mean *Ω*_arag_, demonstrating the importance of algal photosynthesis in the mixed community (Table 2, Fig. 4). Sediment was clearly a source of TA and Ca^2+^ during the course of the experiment (Table 2). The TA increase by the sediment was 12.8 mmol TA m^−2^ d^−1^, which matches rates found by Boucher *et al.* (1998) (Fig. 3). This magnitude of TA flux is only 5% the average TA flux from the mixed-community run. All together the sediment + algae + plankton TA flux signal was about 10–15% of the mixed-community TA signal. The individual non-coral runs showed slight calcification G_net_ (Table 2) due to presence of a limited biomass of calcifying organisms such as crustose coralline algae, barnacles and mollusks.

The algae-only run used the macro-algae, *Gracillaria salicornis,* which is common in Kaneohe Bay. This species is a thick-branched macro-algae that has calcareous epibionts and some crustose coralline algae on the branch bases. The algae showed both a release and uptake in TA over different time intervals on the order of −34.8 to +41 mmol TA m^−2^ d^−1^ (Fig. 4).

The change in TA for the plankton run was very small with a mean of + 5.6 mmol TA m^−3^d^−1^, which is comparable to the magnitude measured in the seawater controls run by Small & Adey (2001).

### Diel cycle

**Figure 5:**
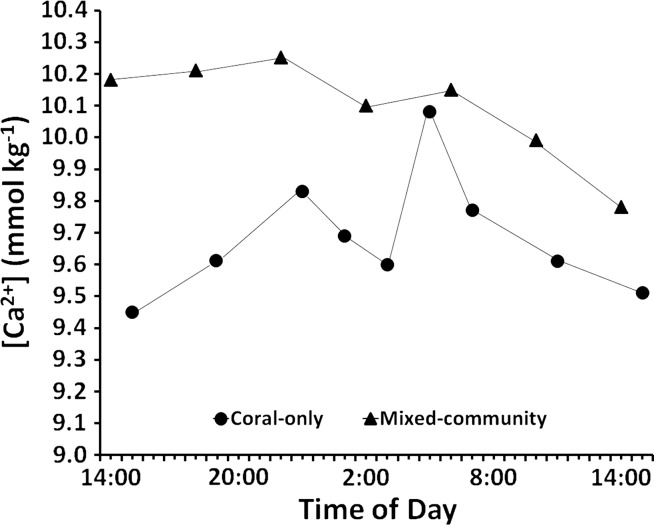
Diel calcium levels. Changes in calcium concentration over 24 h for the coral-only and mixed communities. Overall concentration is higher in the mixed community and there is virtually no net change in calcium concentration at night between 19:00 and 7:00 h. There are higher rates of net dissolution in the coral-only community.

Results of the diel cycle measurements (Fig. 5) show consistently higher [Ca^2+^] in the mixed-community compared to the coral-only community as was the case in the daytime measurements (Table 2). The diel cycle mean [Ca^2+^] for the mixed community (10.08 ± 0.06 mmol kg^−1^) versus the coral-only run (9.68± 0.06 mmol kg^−1^) is similar to that measured during daytime (10.05 ± 0.07 mmol kg^-1^ for the mixed community versus 9.75 ± 0.07 mmol kg^−1^for the coral-only run (Table 2). The diel calcification rates reported in Table 3 were calculated from changes in [Ca^2+^] data for each time interval. Decreases in [Ca^2+^] were considered to represent net dissolution while increases in [Ca^2+^] were taken to represent net calcification. In the coral-only run, average night dissolution was +142 ± 39 *μ*mol Ca^2+^m^−2^ h^−1^. Night dissolution in the mixed community from 19:00 h to 7:00 h was ten-fold lower at +9.57 ± 1.5 *μ*mol Ca^2+^ m^−2^ h^−1^ (the measured 50 *μ*mol increase was averaged over 4 h) *p*-value = 0.053. During the night *Ω*_arag_ increased from the time of sunset to around 11 pm in both the coral-only run and the mixed-community run. A spike in calcification occurred between 11 pm and 1 am as both systems underwent a phenomenon of midnight calcification which has been consistently observed in mesocosm experiments (e.g., Andersson *et al.*, 2009, PL Jokiel, unpublished data). Midnight calcification from 23:00 h to 1:00 h was higher for the coral-only run (−78.8 *μ*mol Ca^2+^ m^−2^ h^−1^) than the mixed community (−44.3 *μ*mol Ca^2+^ m^−2^ h^−1^). From 7 pm to 7 am the net change in calcium concentrations were +164 *μ*mol Ca^2+^(net dissolution) for the coral-only run and −39 *μ*mol Ca^2+^ (net calcification) for the mixed community run.

**Table 3:** Diel calcium flux. Night-time Ca^2+^ flux (calcification and dissolution) comparing coral-only run versus mixed-community run. Table displays measured net change in calcium concentrations. Mean dissolution is the average rate of net dissolution during all nighttime periods. Midnight calcification was between 23:00 and 1:00 h.

	Mean dissolution (**μ**mol m^**−2**^ **h**^**−1**^)	Calcification at midnight (**μ**mol m^**−2**^ **h**^**−1**^)	19:00–07:00 h Net **Δ** Ca^**2+**^ mmol
Coral-only	142 ± 39	78.8	+ 164
Mixed-community	9.57 ± 1.5	44.3	−39
*p*-value	0.053		

## Discussion

### *Δ*TA:*Δ*Ca^2+^ ratio

The measured *Δ*TA:*Δ*Ca^2+^ ratio for the coral community was very close to 2.0 (1.98–2.01), so the alkalinity anomaly technique appears to be valid for coral only experiments and environments dominated by coral cover. Ca^2+^ flux is difficult to measure in the field due to the small changes in concentration over time unless there is sufficiently long residence time and high coral biomass as in these experiments. The *Δ*TA:*Δ*Ca^2+^ ratio was lower than 2.0 (1.55–1.7) when sediments and algae were included with corals, so extra precautions should be used when estimating reef calcification in the field or in mixed community laboratory experiments using the alkalinity depletion method. In systems where corals make up the vast majority of the surface area with a strong hydrodynamic regime (e.g., exposed fore-reef) the use of the *Δ*TA:*Δ*Ca^2+^ ratio = 2.0 may be adequate. However, the method will underestimate gross calcification in lagoons, reef flats, or shallow coastal areas with high sedimentary or algal cover. The potential shortcomings of the alkalinity depletion method were known from the outset (Smith & Kinsey, 1978), and results of this investigation clarify the causes of departure from the theoretical 2.0 value.

The lowered *Δ*TA:*Δ*Ca^2+^ ratios observed in the mixed community can be explained by additional sources of TA from other ecosystem components and processes, especially from the sediment. These sources of TA compensate for the uptake of TA from the water column resulting from calcification. The end result is a lower *Δ*TA:*Δ*Ca^2+^ ratio than would occur if only corals were present.

### Water chemistry conditions

The higher and more stable pH and lower *p*CO_2_ that was observed in the flume when algae and sediment was included with corals support the findings by Thomas *et al.* (2009). Higher pH levels promote CaCO_3_ precipitation (Alongi, Tirendi & Goldrick, 1996). The stable, elevated levels of pH seen in the mixed-community compared to the coral-only community were also highly affected by the presence of the photosynthetic algae.

Anthony, Kleypas & Gattuso (2011) conducted flume experiments on macro-algae communities and coral communities and showed that upstream algal beds elevate *Ω*_arag_ while downstream coral beds decrease *Ω*_arag_. They developed a model based on photosynthesis, respiration, calcification and dissolution coupled with Lagrangian transport show how areas dominated by macroalgae on coral reefs can raise *Ω*_arag_ significantly during the day, effectively improving the conditions for calcification in downstream habitats. The *Anthony et al.* model is supported by results of the present investigation with several additional insights but did not consider the contribution of sediment alone (2011). In their flume, the coral-only run as well as the macro-algae run were established on a 3 cm thick base of reef sand collected from their native habitat. In our studies the sediment-only run resulted in a dramatic increase in TA and higher levels of Ca^2+^ due to dissolution (Eq. (2)), and therefore accounted for the higher TA observed in the mixed-community run (Table 2). The algae raise the pH during daylight hours, which is beneficial to calcification. The sediment increases TA, which also benefits calcification. Anthony, Kleypas & Gattuso (2011) account for the positive effect of photosynthesis, but not the sediment effect.

McConnaughey (2000) investigated the impact of increased pH (lower [H^+^]) on calcification by incubating corals in aquaria with and without macroalgae (sediment was not considered in these experiments). When corals and algae were incubated together, the pH increased (i.e., [H^+^] decreased). Calcification rates increased by 60% for the coral *Acropora* and by 130% in the coral *Montipora* compared to corals incubated without the macroalgae. Accelerated calcification occurred even though [HCO$_{{3}}^{-}$] was diminished by the algae. A similar effect has been noted where high pH values in the field caused by seagrass photosynthesis were shown to enhance calcification rates of associated calcifying macroalgae (Semesi, Beer & Björk, 2009*a*; Semesi, Kangwe & Björk, 2009*b*). Results of the present experiment are consistent with these observations.

Our results are also consistent with the observations of Suzuki, Nakamori & Kayanne (1995), who measured photosynthetic enhancement of calcification in an embayment. Photosynthesis increased pH during the day and calcification rate increased accordingly. They constructed a model that is built on the assumption that the rates of photosynthesis and calcification are proportional to concentrations of their inorganic carbon source. This assumption has recently been challenged by the proton flux hypothesis (Jokiel, 2011*a*; Jokiel, 2011*b*), which maintains that calcification can be limited by rate of H^+^ efflux through boundary layers as well as by DIC concentration. According to this hypothesis the higher pH (lower [H^+^]) and higher TA observed in these experiments would enhance dissipation of waste protons to enhance calcification rate.

From the above discussion it is clear that presence of algae and sediment will increase calcification in reef corals. The alkalinity depletion method will yield a lower net calcification for a mixed-community versus a coral-only community due to TA recycling. The increase in coral calcification for corals growing in the presence of sediment and algae is due to increasing inorganic carbon on the supply side of the calcification equation and accelerating proton removal on the product side of the equation (Eq. (2)) through higher TA and lower [H^+^] on the product side of the equation. The mixed community has a higher *Ω*_arag_ (Table 2) which is generally taken to mean a more favorable environment for coral calcification. Thus a paradox is that corals calcify at a higher rate in a mixed community that will show a lower rate of TA uptake, lower rate of Ca^2+^uptake and a lower *Δ*TA:*Δ*Ca^2+^ ratio (Table 1).

### Diel cycle

The well documented diel cycle of coral reefs (Boucher *et al.*, 1998; Gattuso *et al.*, 1998; Atkinson & Cuet, 2008; Clavier *et al.*, 2008; Shamberger *et al.*, 2011) is the result of connectivity between photosynthesis and respiration with calcification and dissolution. During the day, community primary production increases pH with an increased *Ω*_arag_ and higher rates of CaCO_3_ precipitation (Gattuso *et al.*, 1998; Langdon & Atkinson, 2005) as well as changing the speciation of inorganic carbon into carbonate and bicarbonate ions. Coral and algal photosynthetic and respiratory processes affect the [*p*CO_2_], pH, *Ω*_arag_, and DIC to either enhance calcification or the dissolution processes as seen by Anthony, Kleypas & Gattuso (2011) and Small & Adey (2001). Carbonate dissolution occurs throughout the diurnal cycle, but may accelerate at night during anoxic, low pH conditions. Such dissolution buffers the system and may protect the living corals during periods of very low pH.

Net dissolution of the system is not necessarily indicative of coral decline. Mixed-community systems can undergo net community dissolution while corals within the system show net increases in coral skeletal CaCO_3_ (Andersson *et al.*, 2009). Carbonate sediments and coral rubble played an important role in buffering the ionic concentrations of the community. Thus the sediment-only run showed the highest average Ca^2+^ and TA (Table 2).

### Limitations of extrapolating models

Saturation state correlates with calcification rate within a given system, but values measured in one system cannot always be extrapolated to other systems. Shamberger *et al.* (2011) found the predicted calcification rate for Kaneohe Bay based on a global model developed by Silverman *et al.* (2009) was only 10% of their measured in-situ rates. In our experiments the coral-only community continued to uptake Ca^2+^ and continued to calcify despite *Ω*_arag_ dropping below 1. Also, due to the difference in water chemistry observed between the coral-only and mixed-community incubations, precautions should be taken to extrapolating coral-only laboratory experiments to ecosystem-scale predictions of calcification or health in the face of ocean acidification.

### Need for further studies

This study and previous experiments did not investigate the magnitude of other processes that presumably contribute little to TA such as nutrient transformation. Uptake of NO$_{{3}}^{{-}}$ generates a strong base, OH^−^ whereas NH$_{\mathrm {4}}^{{+}}$ assimilation leads to acid production, H^+^ (Brewer & Goldman, 1976). This means that phytoplankton and algae using nitrate increase TA and those utilizing ammonia generally decrease TA. This study demonstrates that sediments are a source of alkalinity to the water column, which has also recently been shown by Cyronak *et al.* (2013), and may provide local buffering in shallow systems against ocean acidification. TA rapidly increases in sediment with increasing sediment depth where the oxidation and remineralization of organics is occurring (Kuivila & Murray, 1984; Alongi, Tirendi & Goldrick, 1996; Stimson & Larned, 2000; Thomas *et al.*, 2009; Mackenzie *et al.*, 2011). Thus sediments can act as a carbon sink, storing organic matter, and facilitating nutrient cycling, diagenesis and system buffering. (Morse & Mackenzie, 1990; Boucher *et al.*, 1998; Mackenzie & Andersson, 2010; Comeau, Carpenter & Edmunds, 2013). It has been estimated that on a global scale, anaerobic alkalinity generation such as sulphate reduction and denitrification could be accountable for as much as 60% of the uptake of CO_2_ in shelf and marginal seas (Thomas *et al.*, 2009). Thus shallow systems are important drivers of the global carbon cycle which regulates pH and the abundance of CaCO_3_ in the ocean. Sediments and algae cover large areas on coral reefs and are important components of reef metabolism (Werner *et al.*, 2008). A more detailed look at the impact of sedimentary and algal biochemistry on the metabolism of corals would be very beneficial to furthering the understanding of biogeochemical balance on reefs. These components are inherent in coral reef ecosystems and provide feedback loops for calcification and photosynthesis, pH balance, speciation of carbonate ions, nutrient recycling, organic matter oxidation and remineralization. These ecosystem components also provide habitat for a myriad of microorganisms that are crucial to the ecosystem as a whole.

## Summary

∙ Corals isolated from other reef components (sediment and algae) calcified at a 2:1 ratio of TA in respect to Ca^2+^ (*Δ*TA:*Δ*Ca^2+^ = 2.0).
∙ The same corals incubated in a mixed community which included sediment and algae calcified at a lower ratio (*Δ*TA:*Δ*Ca^2+^ = 1.6), which indicates the presence of additional sources of alkalinity (i.e., buffering) from non-coral components of the coral community.
∙ Carbonate sediments incubated in isolation from the other components buffered the water column, maintaining higher and more stable levels of pH while increasing TA and DIC.
∙ Photosynthesis in the macroalgae run raised the pH which provides an environment more favorable to coral calcification.
∙ In these experiments additional TA added by sediment dissolution and algae nutrient transformation represented 10–15% of that consumed by the corals.
∙ The alkalinity depletion method will yield a lower net calcification for a mixed-community versus a coral-only community due to TA recycling, even though the corals may be calcifying at a higher rate due to a more optimal *Ω*_arag_ environment created by the algae and sediment.
∙ The immense map area occupied by sediments and algae on some shallow reefs combined with a low flushing rate could enhance the calcification rate of corals.
∙ Experiments involving isolated coral incubations are crucial to understanding the physiology of organisms. However, precautions should be taken in extrapolating organism-based response to whole-ecosystem response.
∙ Models that predicting future reef growth that are based on data from incubation of isolated corals in would benefit from including the role of carbonate sediments and algae in the design.
∙ Coral reefs with different composition of coral, algae, sediment and other components will have different TA responses, so it is difficult to apply results based on a particular coral reef ecosystem to other reefs.
